# Magnetoelectric Coupling in Ba_0.85_Ca_0.15_Ti_0.92_Zr_0.08_O_3_ with Ultra-Low Concentrations of CoFe_2_O_4_

**DOI:** 10.3390/ma19061243

**Published:** 2026-03-21

**Authors:** Alejandro Campos-Rodríguez, Brayan Carmona-Conejo, Miguel H. Bocanegra-Bernal, Gabriel Rojas-George, Armando Reyes-Rojas

**Affiliations:** Research Center for Advanced Materials, S.C., Miguel de Cervantes 120, Complejo Industrial Chihuahua, Chihuahua 31109, Chihuahua, Mexico

**Keywords:** BCZT-CFO, magnetoelectric, dielectric properties, Rietveld

## Abstract

Magnetoelectric (ME) materials that exhibit simultaneous coupling between electric polarization and magnetization have attracted significant attention due to their potential technological applications in the emerging generation of multifunctional devices. In this research, Ba_0.85_Ca_0.15_Ti_0.92_Zr_0.08_O_3_-CoFe_2_O_4_:x (x = 0.1, 0.2, 0.3% mol) composites were synthesized using solid-state and sol–gel combustion chemical methods to elucidate their ME coupling at ultra-low concentrations of the magnetic phase. Rietveld refinement and Raman spectroscopy results confirm a shift in the morphotropic phase boundary (MPB), evidenced by an increase in the tetragonal phase relative to the orthorhombic structure. High stability of the *P4mm* and *Amm2* symmetries is reached at 1300 °C without diffusion of Fe and Co into the octahedral site. At this temperature, the CoFe_2_O_4_ spinel structure remains stable without secondary phases. The orthorhombic phase fraction decreases from 55% to 37% as the magnetic phase fraction increases, driven by stress and constraint rather than ionic interactions alone. The Curie temperature decreases from 99 to 90 °C, attributed to the grain-size reduction effect rather than structural disorder. The dielectric permittivity (ε_r_) reaches an absolute value of 5070 and progressively decreases with increasing magnetic saturation. An increase in compressive residual stress is observed, which ensures the mechanical stability of the electroceramics. Magnetoelectric (ME) coupling, evaluated through measurements of electric polarization as a function of the magnetic field, shows an increase from 3.8 to 4.9 μC/cm^2^ under a magnetic field of 50 Oe. The composites with x = 0.2 and 0.3 mol% exhibit potential for applications in fast-switching magnetoelectric devices and magnetic field sensors.

## 1. Introduction

Multiferroic materials, which exhibit simultaneous ferroelectric and magnetic properties, have attracted significant attention within the scientific community due to their potential for advanced technological applications, including magnetoelectric sensors, energy harvesters, and next-generation memory devices [1,2,3]. The magnetoelectric (ME) effect, which couples magnetic and electric orders, is particularly attractive because it enables the generation of electric polarization in response to a magnetic field or, conversely, the induction of magnetization under an applied electric field. In general, ME composites consist of two components: a ferroelectric phase and a magnetic phase. The magnetoelectric effect in such composites typically arises from strain-mediated coupling at the interfaces between the piezoelectric and magnetostrictive phases [4].

Lead-free materials have emerged as promising alternatives to conventional piezoelectrics such as lead zirconate titanate (Pb(Zr_1-x_Ti_x_)O_3_, PZT) due to increasing environmental and regulatory concerns. Among these alternatives, barium titanate doped with calcium and zirconium (Ba_0.9_Ca_0.1_Ti_0.9_Zr_0.1_O_3_), BCZT) has shown significant potential for use in lead-free multiferroic composites owing to its excellent piezoelectric properties, high dielectric permittivity, and good stability at room temperature [5]. BCZT ceramics have demonstrated piezoelectric coefficients of up to d_33_ ~600 pC/N and are known for their unique positioning near the polymorphic phase boundary (PPB) between the tetragonal and orthorhombic structures, which enhances their electromechanical performance [6].

For efficient magnetoelectric coupling, the piezoelectric properties of BCZT must be paired with a magnetostrictive phase that exhibits strong magnetoelastic behavior. Magnetostrictive cobalt ferrite (CoFe_2_O_4_) is an ideal candidate due to its high magnetostriction coefficient, excellent chemical stability, mechanical hardness, and high electrical resistivity [7,8]. CFO also exhibits large negative saturation magnetostriction (−252 × 10^−6^), along with a high Curie temperature (T_C_), making it suited for applications at room temperature. Moreover, the high electrical resistivity of CFO helps minimize current leakage, which is critical for maintaining the magnetoelectric effect [9,10]. By combining BCZT and CFO in a composite structure, strain transfer can be optimized through the application of a magnetic field, which induces magnetostrictive deformation in the spinel phase. This strain is subsequently transferred to the BCZT phase, generating an electric polarization response through the piezoelectric effect [11].

In previous studies, BCZT-CFO composites have demonstrated promising performance in multiferroic applications, where the magnetoelectric (ME) coefficient has reached values of approximately159 mV/cm Oe [12], However, further optimization of the ME coefficient remains an important research challenge. Various strategies have been explored to enhance the ME coupling in such composites [13], including optimizing the ratio between the piezoelectric and magnetostrictive phases, tailoring the microstructure, and employing advanced synthesis techniques to improve interfacial bonding [14,15]. Furthermore, reducing the thickness of BCZT pellets has been reported to significantly enhance their piezoelectric performance, as evidenced by increased electrostrain and improved piezoelectric coefficients in reduced-thickness sample [16,17]. In addition, some studies have shown that increasing the sintering time from 2 to 5 h and raising the sintering temperature from 1050 °C to 1300 °C lead to notable improvements in the dielectric constant, magnetization, and ME coupling coefficient of BCZT–CFO composites [7].

Beyond bulk composites, thin-film geometries have also shown significant potential due to their enhanced interfacial mechanical coupling, which enables efficient strain transfer between BCZT and CFO layers. Thin-film heterostructures, such as (Ba(Zr_0.5_Ti_0.5_)O_3_)/(Ba(Ca)TiO_3_/CFO trilayers, can maximize magnetoelectric coupling by improving interface quality and layer thickness control, achieving ME coefficients as high as 0.74 V/cm·Oe [18]. Although thin films may offer superior ME performance, their fabrication complexity and potential stability limitations remain significant challenges. In contrast, bulk BCZT-CFO composites provide a more scalable and stable alternative for practical applications, particularly in devices that require high magnetoelectric response and long-term durability.

Optimizing the balance between piezoelectric and magnetic contributions is essential in BCZT-CFO magnetoelectric systems. Excess magnetic phase disrupts ferroelectric strain-stress tensors, reduces domain mobility, and worsens the piezoelectric response. Using ultra-low concentrations of CoFe_2_O_4_ allows BCZT to maintain its ferroelectric and piezoelectric properties while introducing magnetostrictive domains that support strain-mediated magnetoelectric coupling. This approach is important for developing multifunctional, environmentally friendly magnetoelectric devices.

Our findings are expected to contribute to the growing field of multiferroic materials of ultra-low concentrations of magnetic phase, offering valuable insights into the structure-property relationships that drive magnetoelectric coupling and advancing the potential applications of BCZT-CFO composites in modern electronic devices [15]. Through the study of BCZT-CFO composites with enhanced coupling effects, we strive to support advancements in sensors, data storage, and energy-efficient devices, fostering a transition towards eco-friendly materials in next-generation technology.

## 2. Materials and Methods

Ba_0.85_Ca_0.15_Ti_0.92_Zr_0.08_O_3_-CoFe_2_O_4_:x (x = 0.1, 0.2, 0.3% mol) ceramic composites were prepared using the solid-state reaction method and the combustion synthesis process, respectively. Stoichiometric amounts of BaCO_3_ (99.999%, USA), ZrO_2_ (99.99%, USA), CaCO_3_ (99%, USA), and TiO_2_ (99.8%) were ball-milled with zirconia grinding media in a nylon jar with ethanol for 24 h. The resulting powders were annealed at 1200 °C for 2 h, 1300 °C 12 h, and 1350 °C for 12 h, and 1350 °C for 7 h to promote the formation of the tetragonal and rhombohedral crystal structures. For the synthesis of CoFe_2_O_4_ (CFO) nanoparticles, Cobalt (II) nitrate hexahydrate Co (NO_3_)_2_ 6 H_2_O (99.999%, USA), Iron (III) nitrate hydrate Fe (NO_3_)_3_ 9 H_2_O (99.999%, USA), and Glycine NH_2_CH_2_COOH (99.8%, USA) were used as starting reagents. Stoichiometric quantities of the cation precursors were dissolved in distilled water and stirred for 10 min at room temperature using a hot plate with continuous stirring. The solution temperature was then increased to 80 °C, after which glycine was added as a fuel while the mixture was stirred for 30 min. The resulting gel was dried at 350 °C, and the obtained powders were annealed at 800 °C for 2 h. Subsequently, the BCZT and CFO powders were mixed and ground in an agate mortar for 1 h. The mixed powders were pressed by uniaxial pressing and sintered at 1300 °C for 6 h, using heating and cooling rates of 3 °C/min and 5 °C/min, respectively. Finally, through metallographic procedures, the electroceramic samples were thinned and polished to achieve a diameter-to-thickness ratio of approximately 10:1. Some sample sets were screen-printed with silver epoxy electrodes on both flat surfaces of the discs for electrical characterization.

The crystal structure was analyzed by x-ray diffraction (XRD) using an Empyrean diffractometer equipped with monochromatic radiation (CuKα; λ = 1.54 Å). Crystallographic parameters, as well as the phase fractions of the orthorhombic, tetragonal, and cubic structures, were determined using the Rietveld refinement method implemented in the FullProf software [19]. The surface morphology of the electroceramics was examined using a field-emission scanning electron microscope (FESEM, JSM-7401F, JEOL). The electrical properties were studied from room temperature up to 200 °C at low CFO concentrations using electrochemical impedance spectroscopy (EIS) in a two-electrode configuration, within a frequency range from 0.1 Hz to 100 kHz. Ferroelectric properties were measured using a Precision Multiferroic Analyzer (Radiant Technologies Inc., USA). The magnetic behavior was evaluated through magnetic hysteresis loop measurements using a Physical Property Measurement System (PPMS) equipped with a vibrating sample magnetometer (VSM, Quantum Design).

## 3. Results and Discussion

**Figure 1:**
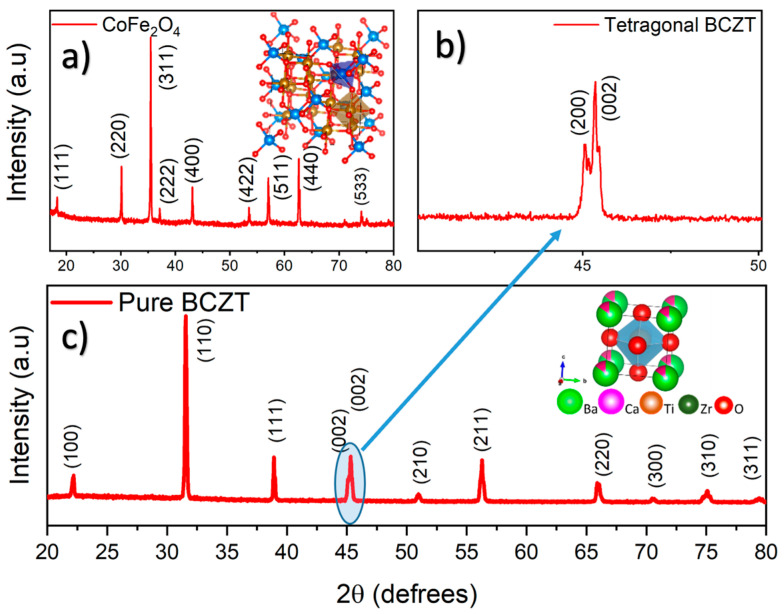
(**a**) XRD of powders of CoFe_2_O_4_ solid-solution with a cubic spinel structure at 1300 °C for 6 h and (**b**,**c**) Ba_0.85_Ca_0.15_Ti_0.92_Zr_0.08_O_3_ solid-solution with tetragonal structure at 1300 °C for 12 h.

To elucidate and confirm the structural features of the composite, an extensive x-ray diffraction (XRD) investigation was undertaken. X-ray diffraction analysis reveals that at 800 °C, the CoFe_2_O_4_ NPs adopt a cubic structure without any secondary phase formation. In order to determine the maximum temperature at which the CoFe_2_O_4_ structure remains stable, the CoFe_2_O_4_ NPs were annealed at temperatures ranging from 800 to 1400 °C. Figure 1a shows the x-ray diffractogram of a powder sample with the spinel-type structure sintered at 1300 °C for 6 h. The *hkl* reflections present in the XRD pattern clearly confirm the formation and stability of the CoFe_2_O_4_ spinel-type structure with the absence of secondary crystals. It should be emphasized that, once this annealing time and temperature are exceeded, the spinel crystal concentration decreases, consequently enhancing the secondary phases. Figure 1c shows an XRD pattern of the Ba_0.85_Ca_0.15_Ti_0.92_Zr_0.08_O_3_ powders annealed at 1300 °C for 12 h. As seen in the figure, the XRD pattern reveals a well-defined tetragonal structure, with no *hkl* reflections corresponding to other phases.

**Figure 2:**
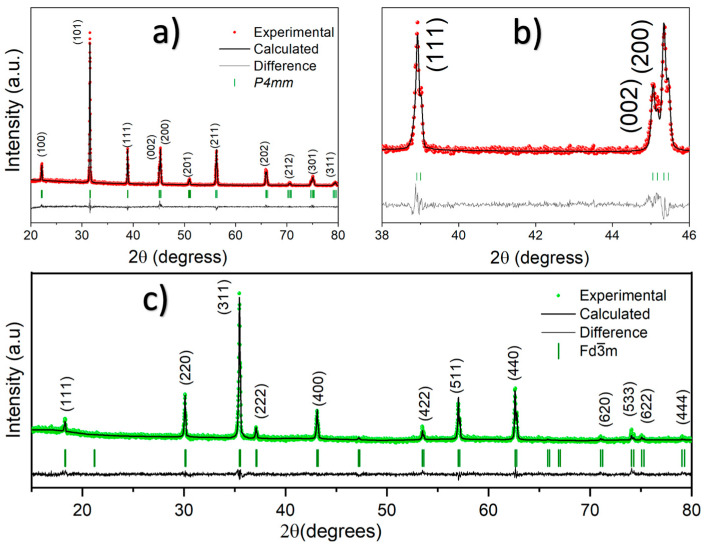
(**a**,**b**) Rietveld refinements of the Ba_0.85_Ca_0.15_Ti_0.92_Zr_0.08_O_3_ solid solution at 1300 °C for 12 h and (**c**) CoFe_2_O_4_ solid solution at 1300 °C for 6 h.

The formation of the tetragonal phase is confirmed by the expanded region of the XRD pattern (two-theta range from 40 to 50 degrees), where the splitting of the (200) and (002) reflections corresponds to the lattice parameters a/b and c of the tetragonal unit cell, respectively (Figure 1b). These results confirm the structural stability of the crystallites under the selected annealing conditions. Figure 2a shows the Rietveld refinement of the X-ray diffraction pattern for Ba_0.85_Ca_0.15_Ti_0.92_Zr_0.08_O_3_ powders annealed at 1300 °C for 12 h. As illustrated in the figure, the Bragg reflections and corresponding (hkl) intensities are in good agreement with the tetragonal symmetry, as evidenced by the close match between the experimental and calculated diffraction patterns. This agreement is further confirmed in the expanded region of the XRD pattern shown in Figure 2b. The diffraction peaks were fitted using a pseudo-Voigt peak shape function, with peak positions and intensities refined according to the P4mm space group. This structure is piezoelectric due to the non-centrosymmetric nature of the 4mm point group. The Rietveld refinement parameters are summarized in Table 1. The occupancy (Occ) of the 1b octahedral site corresponding to Ti/Zr ions is displaced from the origin along the z-axis by 0.4865, producing a local lattice distortion responsible for the development of stable electric dipoles and the resulting piezoelectric behavior [20]. Although the refined lattice parameters (a = 3.9924 Å and c = 4.0163 Å) are close to those of a pseudocubic structure, the non-centrosymmetric tetragonal symmetry is clearly confirmed by the splitting of the (002) and (200) reflections. The quality of the XRD refinement is supported by the conventional agreement indices, with Rp = 10.3 (profile factor) and Rex = 9.96 (expected residual factor), while the goodness-of-fit parameter $\chi^{2}$ is reported in Table 1.

On the other hand, it is known that the spinel form of the CoFe_2_O_4_ can present an inversion degree (i) between the occupancies of the divalent and trivalent cations in the A and B sites of the spinel structure, respectively [21,22]. Thus, depending on the divalent cation concentration on the tetrahedral and octahedral sites, it will induce a distribution of Co and Fe cations with mixed configuration in the solid solution [23]. The inversion degree can be interpreted as follows [24].

(1) $$\left(A_{\left(1-x\right)}^{2+}B_{x}^{3+}\right)\left[A_{x}^{2+}B_{2-x}^{3+}\right]O_{4}$$

where x represents the i, A and B the concertation of Co and Fe ions, respectively.

In this sense, to understand the effect of temperature on the inversion degree of the CoFe_2_O_4_ solid solution, the Rietveld refinement method was applied to the XRD patterns collected at 800 °C and 1300 °C, using the same profile function employed for the perovskite structure. As an example, Figure 2c shows the Rietveld refinement of the spinel structure annealed at 1300 °C for 6 h. The conventional agreement indices were comparable to those obtained for the tetragonal BCZT sample, while the goodness-of-fit parameters ($\chi^{2}$) at 1300 °C and 800 °C were 1.4 and 1.3, respectively (see Table 1). According to the results presented in Table 1, the unit-cell volume does not change significantly with increasing temperature. However, the inversion degree increases from 0.833 to 0.901. Evidently, this behavior depends on temperature, where the interdiffusion of Co and Fe cations into the octahedral and tetrahedral sites, respectively, leads to a more disordered spinel matrix, commonly referred to as a partially inverse spinel structure [25]. Consequently, variations in cation occupancy at the tetrahedral and octahedral sites are expected to influence the charge transport and magnetic properties of the composite [26]. From the Rietveld refinement results, the $Fd\bar{3}m$ symmetry at low temperature exhibits lower disorder, with the 8a site showing approximately 80% occupancy corresponding to tetrahedral-fold coordination (Fe^3+^ concentration). In other words, the stoichiometry tends toward the formation of a $({Co}_{0.167}^{2+}{Fe}_{0.833}^{3+})\left[{Co}_{0.833}^{2+}{Fe}_{1.167}^{3+}O_{4}\right]$ solid solution. In contrast, at high temperature, the occupancy of the 8a site increases to about 90% for tetrahedral-fold coordination, leading to the formation of a $({Co}_{0.099}^{2+}{Fe}_{0.901}^{3+})\left[{Co}_{0.901}^{2+}{Fe}_{1.099}^{3+}O_{4}\right]$ solid solution, with nearly complete inversion. Consequently, the occupancy of the 16d site for the Co^2+^ ion in octahedral coordination increases from 41.65% at low temperature to 45.05% at high temperature.

The inversion degree influences the superexchange interactions responsible for ferrimagnetism. Variations in the distribution of Co^2+^ and Fe^3+^ ions modify the magnetic saturation, magnetocrystalline anisotropy, and magnetostrictive response of the CoFe_2_O_4_ phase. Since the magnetoelectric coupling in Ba_0.85_Ca_0.15_Ti_0.92_Zr_0.08_O_3_-CoFe_2_O_4_:x (x = 0.1, 0.2, 0.3% mol) ceramic composites is strain-mediated, the magnetostriction of the magnetic phase plays a critical role in the efficiency of magnetic-to-electric signal conversion. Thus, changes in the inversion degree may influence the magnetoelectric response by modifying the magnetostrictive strain generated in the CoFe_2_O_4_ phase and its subsequent mechanical transfer to the piezoelectric BCZT matrix [27]. A higher degree of inversion can result in increased magnetocrystalline anisotropy and enhanced magnetostriction, which may improve the strain-mediated magnetoelectric coupling in the composite.

The above results can be explained in terms of point defects according to the Kröger–Vink notation.

At high temperature, the ionic conductivity through the oxygen vacancy

(2) $$8-fold;4-fold\;coordination\;\to {2O}_{O}^{x}\to {2V}_{O}^{{}^{\circ}{}^{\circ}}+4e^{\prime }+O_{2}(g)$$

While at low temperatures, the electronic conductivity increases through cation vacancies.

(3) $${Co}_{Co}^{x}\to V_{Co}^{\prime \prime }+{2h}^{\circ }+Co(s)$$

(4) $${Fe}_{Fe}^{x}\to V_{Fe}^{\prime \prime \prime }+{3h}^{\circ }+Fe(s)$$

However, the defect distribution across tetrahedral and octahedral sites in the CoFe_2_O_4_ structure, where only a few of the atoms in the A site remain, tends to

(5) $$4-fold\;coordination\to {2Fe}_{Co}\to {2Fe}_{Co}^{{}^{\circ}}+2e^{\prime }$$

(6) $$8-fold\;coordination{\to 2Co}_{Fe}\to {2Co}_{Fe}^{\prime }+2h^{{}^{\circ}}$$

The point defects are strongly influenced by the synthesis temperature of the CoFe_2_O_4_ material, and this is a strategy to modify the charge transport and magnetic properties of the spinel structure. The above results are consistent with previous studies by the first-principles calculations [28].

**Figure 3:**
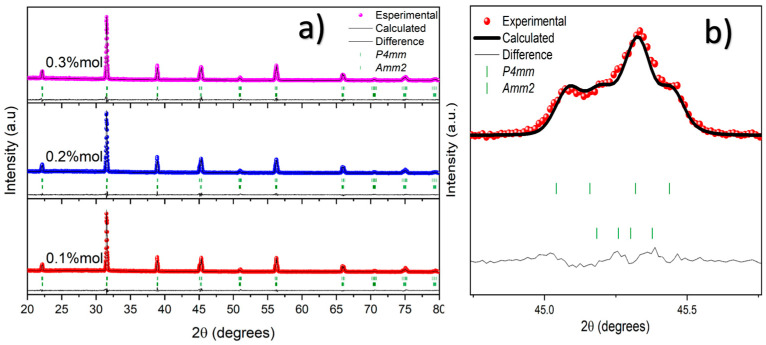
Rietveld refinements of Ba_0.85_Ca_0.15_Ti_0.92_Zr_0.08_O_3_-CoFe_2_O_4_:x composites after sintering at 1300 °C for 6 h. (**a**) BCZT composites with CoFe_2_O_4_ concentrations of x= 0.1, 0.2, and 0.3% mol. (**b**) Enlarged region around ~45 two-theta degrees showing the peak fitting using the P4mm and Amm2 symmetries for x = 0.1.

**Figure 4:**
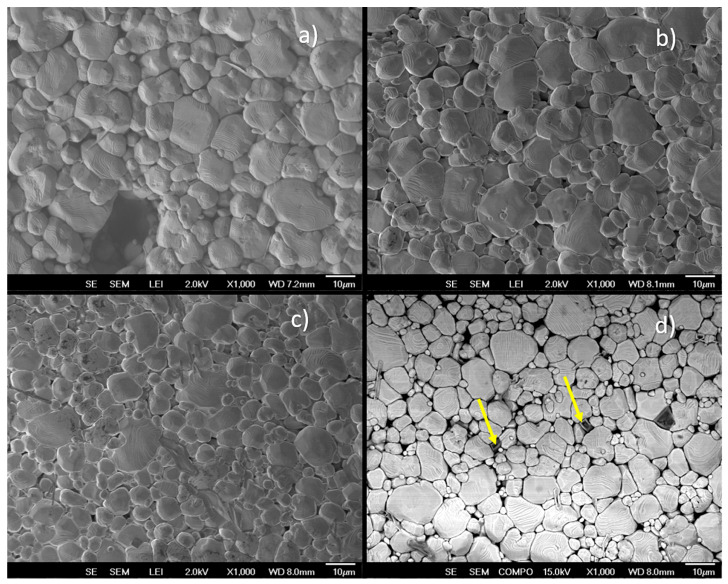
SEM micrographs obtained using secondary electrons of Ba_0.85_Ca_0.15_Ti_0.92_Zr_0.08_O_3_-CoFe_2_O_4_:x composites after sintering at 1300 °C for 6h. (**a**–**c**) show BCZT composites with CoFe_2_O_4_ concentrations of x = 0.1, 0.2, and 0.3 mol%, respectively. (**d**) shows a backscattered electron (BSE) image of the composite with x = 0.3 mol% (arrows represent the CFO particles).

Based on these findings, the CoFe_2_O_4_ powders (annealed at 800 °C for 1 h) were mixed in an agate mortar for 1 h with the Ba_0.85_Ca_0.15_Ti_0.92_Zr_0.08_O_3_ powders (annealed previously at 1300 °C, at 1350 °C for 7 h, and ball-milled in ethanol for 12 h) to form three composites. Figure 3a displays the Rietveld fittings of the Ba_0.85_Ca_0.15_Ti_0.92_Zr_0.08_O_3_-CoFe_2_O_4_:x composites after sintering at 1300 °C for 6 h. The composites with x = 0.1, 0.2, and 0.3% mol are arranged from bottom to top in the figure. The *hkl* reflections of these XRD patterns have been fitted using the tetragonal P4mm and orthorhombic Amm2 symmetries only, without secondary phases. The absence of hkl reflections associated with the CoFe_2_O_4_ cubic phase could be attributed to its extremely low concentration in the composites. The double-phase coexistence in the lead-free multiferroic composites confirms that these composites are at the morphotropic phase boundary (MPB) [29]. Although the molar concentration of ion zircon is slightly lower than that of the commonly studied BCZT [29,30,31], which is 10% compared with 8% in this work, the R3m symmetry does not appear on Ba_0.85_Ca_0.15_Ti_0.92_Zr_0.08_O_3_-CoFe_2_O_4_:x composites, BCZT-CFO. Therefore, the spinel structure could inhibit the formation of the rhombohedral phase during the sintering stage (as mentioned before, the BCZT powders were annealed at 1350 °C for 7 h, after the resulting composites were sintered at 1300 °C for 6 h to ensure structural densification), since in the same experimental condictiones the 0.5Ba(Zr_0.2_Ti_0.8_)O_3_-0.5(Ba_0.7_Ca_0.3_)TiO_3_ ceramics achieved the triple-phase coexistence in tetragonal, orthorhombic and rhombohedral crystal structures [32]. The above can be explained in terms of the interfacial mechanical restriction effect, due to the diffusion of CoFe_2_O_4_ to the grain boundaries and triple junctions of the BCZT matrix. This behavior is evidenced by the SEM analysis (secondary electrons) shown in Figure 4, which reveals that the grain size of BCZT decreases from 12 to 8 µm as the concentration of the magnetic phase increases in the BCZT matrix. Specifically, Figure 4a–c show the magnetoelectric composites with x = 0.01, 0.02, and 0.03 mol%, respectively. To further illustrate the effect, a backscattered electron (BSE) image is included for the electroceramic with x = 0.03 mol% (Figure 4d), providing compositional contrast between different atomic species. In this image, the yellow arrows indicate the presence of the CoFe_2_O_4_ phase, serving as evidence of the interfacial mechanical restriction effect.

Since the elastic modulus and thermal expansion coefficient of CoFe_2_O_4_ differ from those of the BCZT matrix, the CoFe_2_O_4_ grains can influence the microstructural evolution of the BCZT grains. In particular, they may induce compressive stress in the matrix by restricting the growth of BCZT grains. However, the stress–strain state generated in the composites could suppress the formation of the rhombohedral phase, making this phase energetically unfavorable. To support this hypothesis, the results of the Rietveld refinement parameters are analyzed, as summarized in Table 2. The Rietveld refinements of the XRD patterns yielded goodness-of-fit parameters (χ^2^ ≪ 2), confirming the reliability of the fitting, as illustrated in Figure 3b. As expected for tetragonal and orthorhombic structures, the unit-cell volume of the Ba_0.85_Ca_0.15_Ti_0.92_Zr_0.08_O_3_ solid solution remained nearly unchanged after the incorporation of small amounts of the CoFe_2_O_4_ spinel phase. This behavior evidences the high structural stability of the BCZT solid solution and suggests the absence of diffusion of Fe^3+^ and Co^2+^ ions into the octahedral sites of the perovskite lattice. Similarly, the atomic positions of Ti/Zr ions located at the sixfold-coordinated sites are comparable to those observed in powders annealed at 1300 °C for 12 h, further confirming the stability of the tetragonal structure. BCZT has been extensively studied in the composition Ba_0.85_Ca_0.15_Ti_0.92_Zr_0.08_O_3_, in which the tetragonal phase fraction reaches approximately 41% at the morphotropic phase boundary (MPB) [32]. In the present study, the Zr concentration in the solid solution was reduced from 0.10 to 0.08, which results in a slight lattice contraction and promotes greater stabilization of the tetragonal structure. This stoichiometric modification increases the tetragonal phase fraction to 44.99%. Furthermore, with the incorporation of CoFe_2_O_4_, the tetragonal phase fraction increases significantly from 44.99% to 62.73%, indicating a shift in the MPB toward the tetragonal region.

From these results, it is also observed that when the sintering temperature of the composites decreases relative to the annealing conditions (from 1350 °C for 7 h to 1300 °C for 6 h), the morphotropic phase boundary behavior becomes more pronounced. Under these conditions, a partial structural transition from the tetragonal to the orthorhombic phase occurs, driving the electroceramic system toward the tetragonal–orthorhombic (T-O) phase boundary. Consequently, the fraction of the orthorhombic phase decreases from approximately ~55% to about ~37% as the concentration of the spinel phase increases in the composites (see Table 2). This evolution suggests that the incorporation of the spinel phase modifies the structural equilibrium of the BCZT matrix and promotes phase coexistence near the MPB. As a result, the lead-free multiferroic composites exhibit a mixed-phase structure characterized by the coexistence of tetragonal and orthorhombic phases.

**Figure 5:**
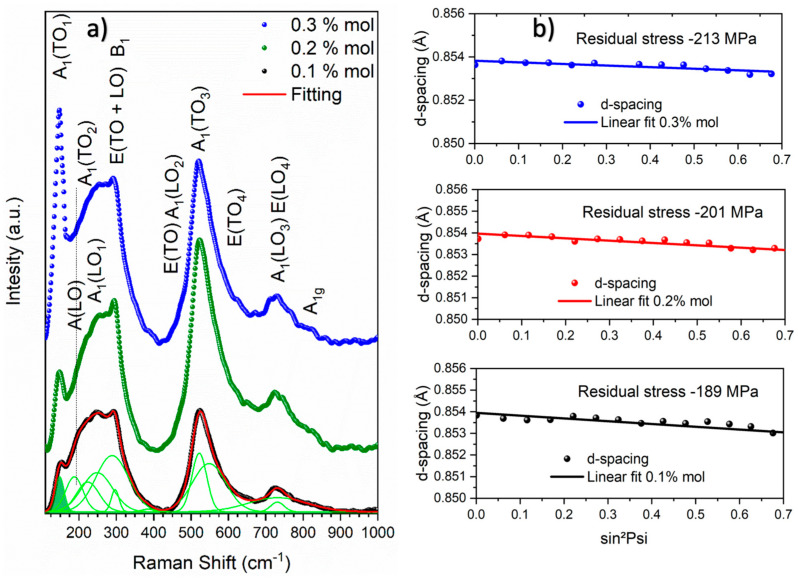
(**a**) Raman spectra of Ba_0.85_Ca_0.15_Ti_0.92_Zr_0.08_O_3_-CoFe_2_O_4_:x (x = 0.1, 0.2, 0.3% mol. The Raman band positions have been fitted and are now shown at the bottom of the figure), and (**b**) the residual stress behavior of these composites.

The above results are consistent with the Raman spectra shown in Figure 5a, which reveal vibrational modes associated with the coexistence of two phases in the lead-free multiferroic BCZT–CFO composites. Nine well-defined polar modes are distinguished in the spectra at approximately ~157, 218, 251, 300, 475, 520, 551, 726, and 810 attributed to A_1_(TO_1_), A_1_(TO_2_), A_1_(LO_1_), E(TO + LO) + B_1_, A_1_(LO_2_) + ETO, A_1_(TO_3_), E(TO_4_), A_1_(LO_3_) + E(LO_4_), and A_1g_, respectively [33,34]. According to group theory, the point group 4mm ($C_{4v}$) exhibits $\Gamma_{C4v}$ = 3[A_1_ (TO) + A_1_ (LO)] + B_1_ + 4 [E(TO) + E(LO)] Raman-active phonon modes [35]. The band at approximately ~300 confirms the presence of the tetragonal structure in the BCZT-CFO composites. This mode is associated with a local octahedral distortion caused by the occupancy ratio of Ti and Zr ions at the 1b site. The corresponding broad signal arises from the asymmetry of the BO_6_ octahedra, which induces local lattice distortion. In addition to these modes, an A(LO) mode observed near 190 cm^−1^ corresponds to the ferroelectric point group mm2 $\left(C_{2\nu }^{14}\right)$ associated with the orthorhombic structure. This vibrational mode has previously been attributed to both orthorhombic and rhombohedral symmetries [36,37,38,39]. However, considering the absence of the rhombohedral phase according to the Rietveld refinement results, this signal can be reasonably assigned to the orthorhombic structure. Furthermore, the asymmetric broadening of the peak near 520 may include a contribution from a mode at approximately 490 cm^−1^, which has been attributed to the orthorhombic phase by other authors [40].

It is well known that BCZT piezoelectric materials exhibit spontaneous electric polarization, which induces high stress concentrations within the crystallographic domains and may generate defects along specific crystallographic directions. These defects within the BCZT microstructure can form parallel dislocations as a consequence of residual stress. At present, reducing piezoelectric losses is crucial, particularly through the immobilization of ferroelectric domain walls [41]. Several strategies have been proposed to improve ferroelectric properties, including doping or the incorporation of secondary-phase particles [42], both of which can regulate the motion of domain walls. The addition of a spinel phase to ferroelectric BCZT at low concentrations is therefore expected to modify its properties. To evaluate the magnitude of the residual stress and its potential influence on the ferroelectric properties, the XRD ${sin}^{2}\varphi$ stress method was applied to the ceramic composites [43], using a maximum value of ${sin}^{2}\psi_{max}=0.7$. Figure 5b shows the $d_{\phi \psi }^{hkl}$ versus ${sin}^{2}\psi$ plots for the Ba_0.85_Ca_0.15_Ti_0.92_Zr_0.08_O_3_-CoFe_2_O_4_:x (x = 0.1, 0.2, 0.3% mol) composites. From the figure, a variation in the *interplanar spacing* of the composites is observed as ${sin}^{2}\psi$ increases, indicating a compressive stress state. The residual stress increases with increasing CoFe_2_O_4_ concentration in the BCZT–CFO composites. A compressive stress of −189 MPa is obtained for x = 0.1 mol%, which increases monotonically to −201 and −213 MPa for x = 0.2 and 0.3 mol%, respectively. As expected, the addition of low concentrations of the spinel phase in 0–3 type magnetoelectric composites increases the residual stress due to the segregation of CoFe_2_O_4_ at the grain boundaries and triple junctions of the BCZT grains. However, the stress component remains compressive, which is beneficial for maintaining the mechanical integrity of the ceramic composites. Compressive stress improves fatigue strength, whereas tensile stress tends to lead to crack formation. In contrast to BCZT thin films [44], where larger tensile stresses are typically reported, the values obtained here remain compressive and therefore do not compromise the structural integrity of the ceramics. It is also worth noting that the bulk density of the electroceramics remains nearly constant with increasing spinel phase content, indicating that the incorporation of this magnetostrictive phase does not significantly affect the densification process. The measured densities were 5.514, 5.502, and 5.497 g/cm^3^ for 0.1, 0.2, and 0.3 mol%, respectively.

**Figure 6:**
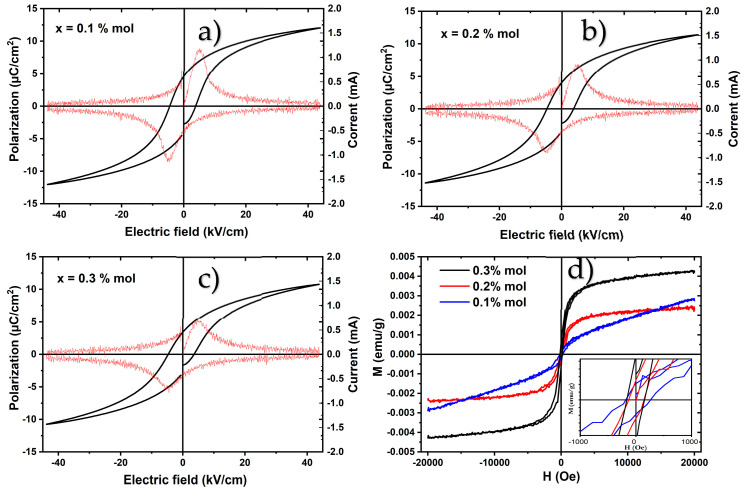
(**a**–**c**) Ferroelectric (the current variation (mA) as a function of the applied electric field is represented by the red curve) and (**d**) magnetic hysteresis loops of Ba_0.85_Ca_0.15_Ti_0.92_Zr_0.08_O_3_ with CoFe_2_O_4_ at x = 0.1, 0.2, 0.3% mol.

Figure 6a–c shows the ferroelectric hysteresis loops of the composites measured at 20 Hz under an electric field of 50 kV cm^−1^. The electroceramics exhibit characteristic P–E loops typical of BCZT ceramic samples, indicating a soft ferroelectric behavior [11]. As expected, a reduction in the ferroelectric properties is observed with the incorporation of the magnetic phase into the composites. For all three compositions, well-saturated hysteresis loops were obtained. At x = 0.1 mol% of the magnetic phase, the saturation polarization (Ps) reaches 12.03 µC/cm^2^, while the remanent polarization (Pr) and coercive field (Ec) are 4.63 µC /cm^2^, and 4.49 kV/cm, respectively. For x = 0.2 and 0.3 mol%, the ferroelectric parameters decrease progressively, with Ps and Pr values of 11.38 µC/cm^2^ and 4.07 µC/cm^2^, and to 10.76 µC/cm^2^ and 3.44 µC/cm^2^, respectively. These results are consistent with the slight decrease in density associated with the incorporation of the CoFe_2_O_4_ spinel structure [38]. However, the coercive field shows an opposite trend with increasing magnetic phase content. The coercive field increases from 4.49 to 4.59 and 4.66 kV/cm for x = 0.1, 0.2, and 0.3 mol%, respectively, indicating that the ferroelectric composites become progressively harder and that greater energy is required to switch the electric domains. It is also evident that the coexistence of tetragonal P4mm and orthorhombic Amm2 symmetries preserves the ferroelectric character of the compounds. Nevertheless, the incorporation of the spinel phase with $Fd\bar{3}m$ symmetry may increase the switching energy barrier; in other words, the free-energy difference required for domain polarization reversal becomes larger [45]. This behavior has commonly been attributed to increased leakage current in 0–3 type magnetoelectric composites, which can influence the polarization switching process [2].

Figure 6d shows the magnetization versus magnetic field curves obtained at room temperature for the composites. At a low CoFe_2_O_4_ concentration, the hysteresis loop exhibits very weak magnetic behavior, resembling an antiferromagnetic-like response. The absence of magnetic saturation can be attributed to the low concentration of the magnetic phase and to interfacial disorder caused by the dilution of the spinel phase within the composite. Consequently, the ferroelectric BCZT matrix dominates the overall magnetic response. However, as the concentration of the spinel phase increases in the composites to x = 0.2 and 0.3 mol%, magnetic saturation (Ms) is reached, indicating the emergence of a ferromagnetic state. In this case, the saturation magnetization increases from 2.4 × 10^−3^ to 4.3 × 10^−3^ emu/g, for x = 0.2 and 0.3 mol%, respectively. Meanwhile, the coercive magnetic field (Mc) increases from 148 to 155 Oe for x = 0.2 and 0.3% mol, respectively (see the inset of Figure 6d). The increase in magnetization is mainly attributed to the higher content of the magnetic phase in the composites. This behavior is consistent with previous studies reported by other authors for similar magnetoelectric composite systems [46,47].

**Figure 7:**
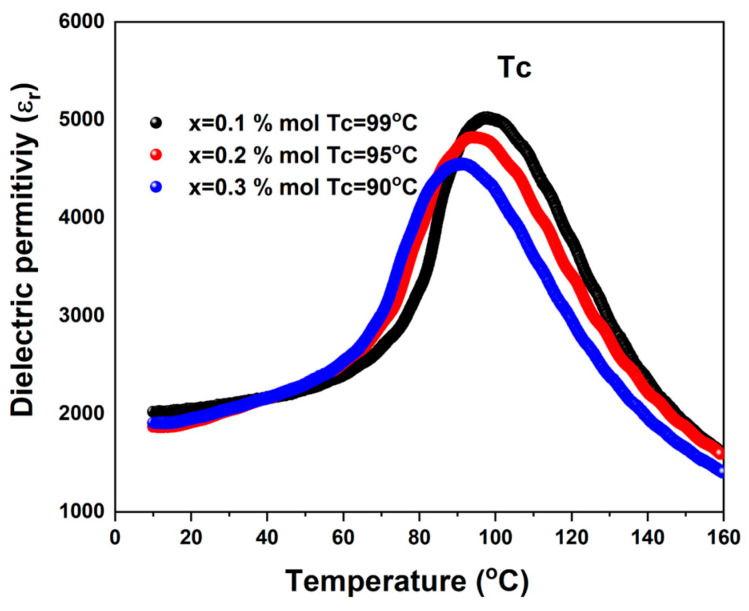
Dielectric permittivity as a function of temperature measured at 1 kHz for Ba_0.85_Ca_0.15_Ti_0.92_Zr_0.08_O_3_ with CoFe_2_O_4_ composites with x = 0.1, 0.2, and 0.3% mol.

The variation in the dielectric permittivity of BCZT-CFO composites sintered at 1300 °C for 6 h, measured at 1 kHz as a function of temperature, is shown in Figure 7. The samples exhibit broad signals associated with the tetragonal-cubic symmetry transition [48], and tetragonal-orthorhombic around 40 °C. The Curie temperature (T_C_) tends to decrease as the magnetic phase increases. The T_C_ values for the spinel concentrations at x = 0.1, 0.2, and 0.3%mol, were 99 °C, 95 °C, and 90 °C, respectively. These T_C_ values are in agreement with those reported for BCZT ceramics modified by the addition of Nb^5+^ ion and Zn/Ta [36,49], which could be attributed to an increase in the structural disorder, influenced by the change in the tolerance factor due to the ionic radius of these cations [50]; however, in our results, there is no evidence of nucleation of Co or Fe ions at the A or B sites of the perovskites; thus, it has been attributed to the grain-size reduction effect of BCZT crystallites [51,52], where the SEM analysis has found CoFe_2_O_4_ at the grain border of BCZT grains (results shown in Figure 4d). As a consequence, the absolute value of the dielectric permittivity decreases from 5070 to 4593; thus, the insulating properties of the dielectric material are affected.

According to the previous results on polarization and magnetization, it is evident that the observed properties of the composites arise not from their chemical composition alone but from the intrinsic ferroelectric and magnetic characteristics of BCZT and CoFe_2_O_4_ (CFO), respectively. To determine whether magnetoelectric (ME) coupling exists in the composites, measurements were performed using Radiant equipment equipped with a Magnetoelectric Response Bundle. The samples were measured in the transverse configuration, where the electric polarization vector P is perpendicular to the magnetization vector M. In this configuration, the magnetoelectric coupling response is described by the ${ME}_{31}$ form. The magnetic field was varied slowly while the polarization response of the composite was recorded, allowing the estimation of the magnetoelectric coupling through the change in polarization with respect to the applied magnetic field. The ME coupling is mainly measured in voltage (E field) form as a response to an AC magnetic field (H) applied [53], where the ME coefficient α is obtained as follows

$$\alpha_{MN}=\frac{\partial E}{\partial H_{ac}}$$

where E is the electric field–induced signal measured across the sample, and H is the applied AC magnetic field. However, in the Magnetoelectric Response System from Radiant, a virtual ground is connected across the capacitor; therefore, the magnetoelectric coefficient α is measured under zero applied electric field conditions.

$$\alpha =\frac{P}{H}$$

and the ME coefficient $\alpha_{ME}$

$$\alpha_{ME}=\frac{\alpha }{\varepsilon_{o}\varepsilon_{r}}$$

where the P, H, $\varepsilon_{o}$, and $\varepsilon_{r}$ elements are the electric polarization, magnetic field, vacuum permittivity, and material permittivity, respectively [54].

**Figure 8:**
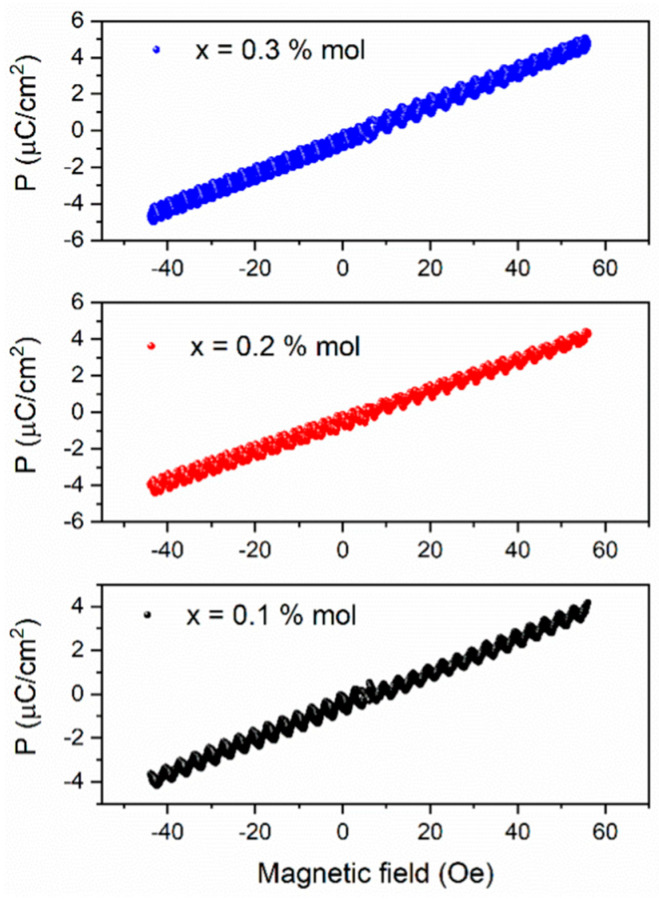
Magneto-electric coupling through electric polarization as a function of magnetic field for Ba_0.85_Ca_0.15_Ti_0.92_Zr_0.08_O_3_ with ultra–low additions of CoFe_2_O_4_ (**a**) x = 0.1% mol. (**b**) x = 0.2% mol., and (**c**) x= 0.3% mol.

The ME effect is shown in Figure 8, where the variation in a magnetic field applied induces an electric polarization to the Ba_0.85_Ca_0.15_Ti_0.92_Zr_0.08_O_3_-CoFe_2_O_4_:x (x = 0.1, 0.2, 0.3%mol) composites. From the figure, the polarization variation exhibits a linear behavior with the applied magnetic field. At 0.1%mol of CoFe_2_O_4_ in the Ba_0.85_Ca_0.15_Ti_0.92_Zr_0.08_O_3_ perovskite reaches a polarization of 3.8 μC/cm^2^, with a magnetic field of 50 Oe, while for x = 0.2 and x = 0.3 the composites reach 4.2 and 4.9 μC/cm^2^, respectively. Obviously, this relationship implies that it is also possible to modify the magnetic dipoles of the composite by applying an electrical polarization. As expected, the magnetic field induces a strain component in the magnetostrictive CoFe_2_O_4_ spinel structure, which deforms to the Ba_0.85_Ca_0.15_Ti_0.92_Zr_0.08_O_3_ piezoelectric, thus inducing an electric polarization.

It is well known that in magnetoelectric composites with 0–3 phase connectivity based on magnetostrictive phases such as CoFe_2_O_4_ combined with piezoelectric materials such as Ba_0.85_Ca_0.15_Ti_0.92_Zr_0.08_O_3_, the ME coupling reflects the efficiency of polarization change induced by a magnetic field facilitated by elastic strain transfer between the constituent phases [7]. Therefore, the observed increase suggests improved mechanical coupling between the phases and a stronger interaction between magnetostriction and piezoelectricity.

From an application perspective, higher ME coefficients are desirable for devices such as magnetic field sensors, energy harvesting systems, and multifunctional transducers, where the sensitivity and efficiency of magnetic-to-electric signal conversion are critical. Consequently, the improvement ME coupling response reported in this work may contribute to enhanced device performance in low-field magnetic sensing and energy conversion applications.

Based on these findings, it is demonstrated that the incorporation of ultra-low concentrations of CoFe_2_O_4_ with a spinel structure into BCZT piezoelectric ceramics promotes a significant magnetoelectric coupling, which could enable potential applications in fast-switching ME devices and magnetic field sensors.

## 4. Conclusions

This study investigated the dielectric, ferroelectric, and magnetoelectric properties of Ba_0.85_Ca_0.15_Ti_0.92_Zr_0.08_O_3_:x (x = 0.1, 0.2, 0.3% mol) composites with 0–3 phase connectivity (particulate composites), synthesized by solid-state reaction and sol–gel combustion methods. The addition of the spinel phase in sintered composites at 1300 °C leads to a reduction in the absolute value of the dielectric permittivity from 5070 to 4593 and an increase in magnetic saturation. This behavior is mainly attributed to the grain-size reduction effect of BCZT crystallites and to phase dilution effects resulting from the decrease in the piezoelectric phase concentration. Rietveld refinement results indicate that, at high temperatures, the spinel structure exhibits an almost complete inversion degree (i = 0.901), which influences the magnetic saturation. The coexistence of tetragonal and orthorhombic structures is observed at 1350 °C. Furthermore, the Rietveld analysis confirms the high structural stability of both BCZT and CFO crystal structures, with no evidence of Fe^3+^ and Co^2+^ ion diffusion into the octahedral sites of the perovskite lattice. The residual stress component increases with increasing spinel phase concentration, which has been interpreted in terms of cobalt-ferrite diffusion to the BCZT matrix, restricting BCZT grain growth due to the CFO volume fraction.

Analysis of the ferroelectric properties through P–E hysteresis loops shows that saturation polarization (Ps) decreases from 12.03 µC/cm^2^ to 10.76 µC/cm^2^ with increasing magnetic saturation (Ms) as the CoFe_2_O_4_ concentration increases in the composites.

The magnetoelectric (ME) coupling was confirmed in BCZT–CFO composites with ultra-low concentrations of the spinel phase through the variation in electric polarization under an applied magnetic field. A maximum polarization of 4.9 µC/cm^2^ was achieved at a magnetic field of 50 Oe.

## Figures and Tables

**Table 1:** Structural parameters obtained from Rietveld refinement of Ba_0.85_Ca_0.15_Ti_0.92_Zr_0.08_O_3_ and CoFe_2_O_4_ powders.

Symmetry	Parameters						
**S. G.**	**a, b**	**c**	**Vol. (Å^3^)**	**Ti/Zr (z)**	$\chi^{2}$	**R_p_**	**R_wp_**
*P4mm*	3.9924 (1)	4.0163 (1)	64.017 (1)	0.4865 (1)	1.8	5.02	7.13
*Fd* $\bar{3}$ *m_800C_*	8.3848 (1)		589.51 (1)		1.3	3.21	4.17
*Fd* $\bar{3}$ *m_1300C_*	8.3852 (1)		589.59 (1)		1.4	4.13	5.32
*Inversion degree I* = 0.833 at 800 °C *I* = 0.901 at 1300 °C							

The number in parentheses represents the uncertainty in the last digit.

**Table 2:** Rietveld refinement parameters for Ba_0.85_Ca_0.15_Ti_0.92_Zr_0.08_O_3_-CoFe_2_O_4_ composites after sintering process at 1300 °C for 6 h.

Parameter	S.G.	Composition (% mol)		
		0.1	0.2	0.3
a	*P4mm*	3.9966 (1)	3.9977 (1)	3.9959 (1)
b		3.9966 (1)	3.9977 (1)	3.9959 (1)
c		4.0174 (1)	4.0153 (2)	4.0191 (1)
Vol. (Å^3^)		64.172 (1)	64.174 (1)	64.177 (1)
Ti (*z*)		0.5023 (1)	0.5021 (1)	0.4845 (1)
Conc. (%)		44.99 (1)	52.90 (1)	62.73 (1)
a	*Amm2*	4.0038 (1)	4.0034 (1)	4.0012 (1)
b		5.6743 (1)	5.6790 (1)	5.6787 (1)
c		5.6681 (1)	5.6709 (1)	5.6619 (1)
Vol. (Å^3^)		128.777 (1)	128.935 (1)	128.651 (1)
Conc. (%)		55.01 (1)	47.10 (1)	37.27 (1)
R_p_		2.91	2.06	2.63
R_wp_		3.77	2.74	3.37
$\chi^{2}$		1.47	1.51	1.59

The number in parentheses represents the uncertainty in the last digit.

## Data Availability

The original contributions presented in this study are included in the article. Further inquiries can be directed to the corresponding author.
